# Virus detection in *Culicoides* biting midges during the BTV-3 outbreak in north–west Germany 2024

**DOI:** 10.1186/s13071-026-07767-6

**Published:** 2026-10-30

**Authors:** Sophie Zeiske, Helge Kampen, Dorothee Scheuch, Franziska Sick, Martin Beer, Anja Voigt, Doreen Werner, Kerstin Wernike

**Affiliations:** 1 Friedrich-Loeffler-Institut, Federal Research Institute for Animal Health, Greifswald-Insel Riems, Germany; 2 Leibniz-Centre for Agricultural Landscape Research, Muencheberg, Germany

**Keywords:** Bluetongue virus, Schmallenberg virus, *Culicoides*, Biting midges, Monitoring study

## Abstract

**Background:**

Bluetongue virus (BTV), Schmallenberg virus (SBV) and epizootic hemorrhagic disease virus (EHDV) are arthropod-borne pathogens that are transmitted by biting midges of the genus *Culicoides*. Following the initial introduction of BTV in central Europe in 2006, the emergence of SBV in 2011, and the first detection of EHDV in southern Europe in 2022, it became evident that *Culicoides*-borne viruses pose a significant threat to European livestock. In 2023, BTV serotype 3 was detected for the first time in the Netherlands and subsequently caused a severe outbreak in Germany in 2024. The aim of this study was to compare the prevalences of the viruses in the *Culicoides* population during the BTV-3 outbreak in north–west Germany in 2024.

**Methods:**

*Culicoides* were caught by UV-light traps on 14 farms from April to October 2024 in north–west Germany. 337,359 of those were selected for virus screening and sorted into 7,549 pools based on their morphology. The pools were tested by real-time RT-PCRs for the genomes of SBV, BTV and EHDV. Virus-positive pools were further analyzed in species-specific PCRs to identify the *Culicoides* species present.

**Results:**

A total of 1,359 pools (18.0%) tested positive for BTV–RNA, while 402 pools (5.3%) showed SBV–RNA. None of the pools were positive for EHDV. The majority of the positive pools contained specimens of the *Culicoides* subgenus *Avaritia* Fox. A smaller part belonged to the subgenus *Culicoides* Latreille. The taxa most commonly found in the virus-positive pools of the subgenus *Avaritia* were *C. obsoletus* clade O1, *C. scoticus* and *C. dewulfi.* The most prevalent taxa in the virus-positive pools of the subgenus *Culicoides* were *C. punctatus, C. pulicaris and C. lupicaris* haplotype L1*.*

**Conclusions:**

We confirmed the ongoing circulation of SBV as well as an extensive BTV circulation in the *Culicoides* vectors of the subgenera *Avaritia* and *Culicoides* in north–west Germany in 2024. Despite belonging to different virus families, the circulation patterns of BTV and SBV observed over the months were very similar, showing peaks in August.

**Graphical Abstract:**

**Figure:**
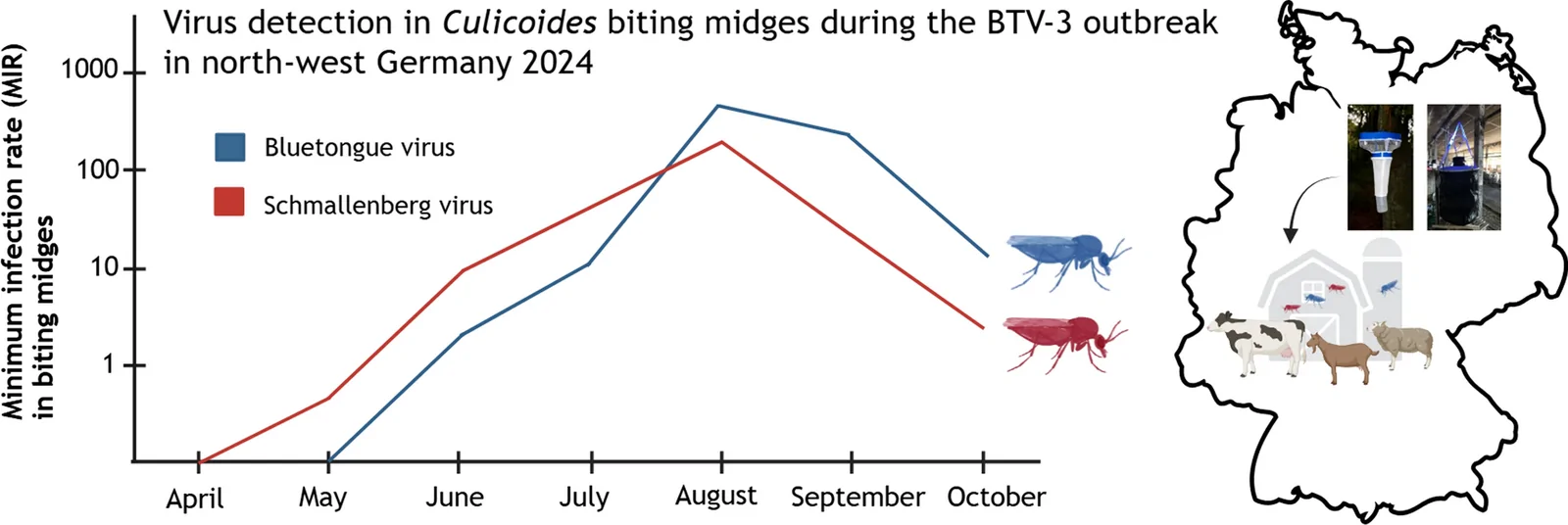

## Background

Schmallenberg virus (SBV), bluetongue virus (BTV) and epizootic hemorrhagic disease virus (EHDV) are pathogens transmitted by biting midges and predominantly affect ruminants. Biting midges of the genus *Culicoides* belong to the family Ceratopogonidae within the insect order Diptera and are approximately 2–4 mm in length [1]. The females are haematophagous and can be vectors of pathogens of animals and humans [1]. The viruses transmitted by *Culicoides* in Europe only affect animals, primarily ruminants. In northern and central Europe, six species of *Culicoides* have been implicated in the transmission of BTV, EHDV and SBV; *Culicoides obsoletus* (Meigen), *Culicoides scoticus* Downes & Kettle, *Culicoides chiopterus* (Meigen) and *Culicoides dewulfi* Goetghebuer, from the subgenus *Avaritia* Fox, 1955, and *Culicoides pulicaris* (Linnaeus) and *Culicoides punctatus* (Meigen), from the subgenus *Culicoides* Latreille, 1809 [2–12]. These species, along with other members of the subgenus *Culicoides*, are the most abundant across livestock-associated habitats [13, 14].

Vector competence is the ability of an arthropod vector to replicate and transmit a virus during a blood meal [15]. It requires complete viral dissemination and replication in the salivary glands [16]. In *Culicoides*, vector competence is typically assessed by examining the body and head separately; virus detection in the head is considered sufficient evidence of dissemination and infectivity, as biting midges lack salivary gland barriers [15, 16]. The mere detection of viral genetic material in *Culicoides* does not necessarily implicate vector competence.

The orthobunyavirus SBV was first identified in Germany in 2011, and has since spread throughout Europe [17, 18]. SBV mainly affects domestic ruminants, such as cattle, sheep and goats, though it has also been serologically detected in wild ruminants, such as roe deer, red deer and mouflons [17, 19–21]. The acute infection of adult animals is mostly asymptomatic. In some cases, infected ruminants exhibit mild clinical signs such as fever, diarrhea, or a decline in milk production [17, 22, 23]. However, SBV infection of a naïve pregnant dam in the early stages of gestation can lead to premature birth, stillbirth, abortion, or fetal malformation [24, 25].

BTV and EHDV belong to the *Orbivirus* genus and can be differentiated in several serotypes. More than 25 serotypes of BTV were detected in various combinations on every continent, except for the polar regions of Antarctica [26]. The seven serotypes of EHDV are similarly widespread and were detected in North and South America, Asia, Australia, Africa and southern Europe [12, 27, 28]. Both BTV and EHDV cause diseases in domestic and wild ruminants; however, the clinical manifestation of the infection depends on various factors, such as the species infected, the viral serotype and particular strain [12, 29]. Since 2023, there has been an ongoing outbreak of BTV-3 in northern Europe, including Germany, which has caused severe disease in both sheep and cattle [30, 31]. In 2022, EHDV-8 was detected for the first time in Europe [28, 32] and caused an outbreak in France in 2023 [27]. EHDV-8 was detected in sheep and cattle, though symptomatic infections were observed mainly in cattle [27]. Only a limited amount of data are available on the infection of wild ruminants with EHDV-8 in France. However, the testing of four spleens of Pyrenean chamois, roe deer and red deer, that were found dead, revealed a positive result for EHDV [27]. So far, no cases of EHDV have been detected in ruminants in Germany. BTV and EHDV cause haemorrhagic fevers, and the clinical signs can comprise edema, hyperemic and ulcerative lesions of the facial mucosa and lameness due to lesions of the coronary band [33, 34]. In severe cases, the viruses can affect internal organs, leading to the death of infected animals [35]. Similar to SBV, infection of pregnant dams with BTV or EHDV can result in stillbirth, abortion or fetal malformation [36].

Surveillance of *Culicoides*-borne viruses in Europe is important from a veterinary perspective, because the diseases they spread threaten animal welfare and the productivity of livestock farms. In October 2023, BTV-3 was detected for the first time in western Germany; however, at that time, the virus still circulated at a low level [31, 37].

The aim of this study was to monitor the prevalence of viruses in native *Culicoides* species in north–west Germany, focusing on the prevalence of BTV within the *Culicoides* population as the BTV-3 outbreak progressed throughout 2024. For this purpose, *Culicoides* were caught on livestock farms, morphologically identified to subgenus level, pooled and tested for the genomes of SBV, BTV and EHDV. This approach enabled a comparison to be made between the prevalence of SBV, which is enzootic in Germany, and BTV, which is emerging. In addition, testing for EHDV was conducted due to its presence in neighboring France.

## Methods

**Fig. 1:**
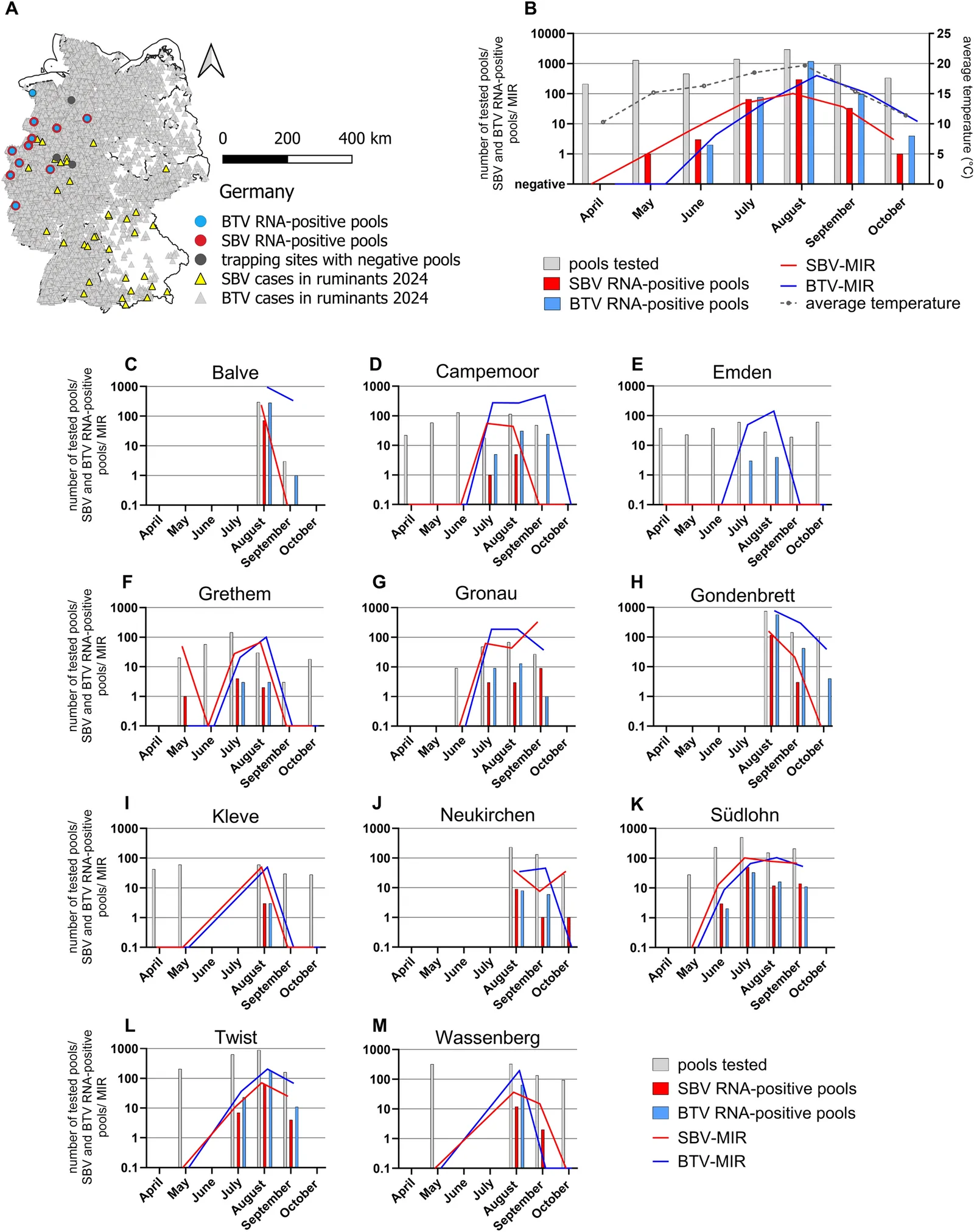
Location of *Culicoides* trapping sites and results of virus screening in the *Culicoides* pools. The locations of the trapping sites are illustrated in (**A**) with gray dots depicting sites with no virus detection, blue dots with bluetongue virus (BTV) RNA detection and red dots with Schmallenberg virus (SBV) detection. The gray triangles in **A** represent cases of BTV, the yellow triangles cases of SBV in ruminants reported to the German animal disease reporting system TSN in 2024. The monthly number of tested pools of *Culicoides* (gray bar), the number of BTV–RNA-positive pools (blue bar) and the number of SBV–RNA-positive pools (red bar) for all monitored sites in 2024 are depicted in (**B**) and for each individual trapping site in (**C**–**M**). In **B**, the dotted gray line represents the average monthly temperature in the monitored federal states [49]. To show monthly differences of virus circulation, the minimum infection rate (MIR) was calculated for BTV (blue line) and for SBV (red line) in (**B**–**M**)

The present *Culicoides* monitoring programme in Germany was established in 2019, and the methods used for *Culicoides* trapping, identification and nucleic acid extraction as well as for virus screening have been published in detail [37]. In this study, the *Culicoides* were collected on 14 farms keeping cattle, sheep or goats in the north-western German federal states of North Rhine–Westphalia, Rhineland–Palatinate and Lower Saxony, where BTV-3 was expected to emerge first in Germany after its appearance 2023 in the Netherlands (Fig. 1, Table 1). The *Culicoides* were trapped by UV-light BG-Sentinel traps (Biogents AG, Regensburg, Germany). Each farm was equipped with one trap, which was placed either inside or outside of the farm buildings. Collections were conducted daily from April to October 2024. All caught insects were stored in 80% ethanol and kept in the dark at room temperature until further processing, in order to preserve morphological features and minimize degradation of nucleic acids.

**Table 1:** Results of *Culicoides* screening according to month and location

Trapping site	German federal state	Ruminants kept	April	May	June	July	August	September	October	Total
Balve	North Rhine–Westphalia	Cattle	−	−	−	−	297 (14,445) B: **283**, S: **71**	3 (41) B: **1**, S: 0	−	300 (14,486) B: **284**, S: **71**
Gronau	North Rhine–Westphalia	Cattle	−	−	9 (358) B: 0, S: 0	48 (1,875) B: **9**, S: **3**	69 (2,835) B: **13**, S: **3**	27 (905) B: **1**, S: **9**	−	153 (5,973) B: **23**, S: **15**
Kleve	North Rhine–Westphalia	Cattle	42 (964) B: 0, S: 0	60 (1,734) B: 0, S: 0	−	−	60 (1,119) B: **3**, S: **3**	30 (226) B: 0, S: 0	28 (197) B: 0, S: 0	220 (4,240) B: **3**, S: **3**
Marsberg	North Rhine–Westphalia	Cattle	29 (1,357) B: 0, S: 0	−	−	−	−	−	−	29 (1,357) B: 0, S: 0
Neukirchen	North Rhine–Westphalia	Goat	−	−	−	−	230 (9,516) B: **8**, S: **9**	131 (5,167) B: **6**, S: **1**	28 (943) B: 0, S: **1**	389 (15,626) B: 14, S: 11
Soest	North Rhine–Westphalia	Cattle	81 (4,010) B: 0, S: 0	267 (13,010) B: 0, S: 0	−	−	−	−	−	348 (17,020) B: 0; S: 0
Südlohn	North Rhine–Westphalia	Cattle	−	28 (1,293) B: 0, S: 0	231 (11,352) B: **2**, S: **3**	499 (24,605) B: **33**, S: **51**	153 (7,102) B: **16**, S: **12**	208 (9,698) B: **11**, S: **14**	−	1,119 (54,050) B: **62**, S: **80**
Wassenberg	North Rhine–Westphalia	Cattle	−	321 (14,280) B: 0, S: 0	−	−	328 (13,568) B: **64**, S: **12**	134 (4,931) B: 0, S: **2**	94 (2,753) B: 0, S: 0	877 (35,532) B: **64**, S: **14**
Campemoor	Lower Saxony	Cattle	22 (862) B: 0, S:0	58 (2,801) B: 0, S:0	129 (6,247) B: 0, S: 0	18 (790) B: **5**, S: **1**	114 (5,489) B: **31**, S: **5**	48 (2,187) B: **24**, S: 0	−	389 (18,376) B: **60**, S: **6**
Emden	Lower Saxony	Sheep	37 (1,582) B: 0, S:0	23 (842) B: 0, S:0	37 (701) B: 0, S: 0	60 (993) B: **3**, S: 0	28 (671) B: **4**, S: 0	19 (565) B: 0, S: 0	61 (1,972) B: 0, S: 0	265 (7,326) B: **7**, S: 0
Grethem	Lower Saxony	Sheep	−	20 (866) B: 0, S: **1**	57 (2,731) B: 0, S: 0	144 (6,861) B: **3**, S:** 4**	30 (1,192) B: **3**, S:** 2**	3 (107) B: 0, S: 0	18 (555) B: 0, S: 0	272 (12,312) B: **6**, S: **7**
Osterholz	Lower Saxony	Cattle	−	305 (15,241) B: 0, S: 0	−	−	−	−	−	305 (15,241) B: 0, S: 0
Twist	Lower Saxony	Cattle	−	206 (10,176) B: 0, S: 0	−	633 (30,862) B: **23**, S: **7**	891 (42,319 B: **183**, S: **63**	161 (6,352) B: **11**, S: **4**	−	1,891 (89,709) B: **217**, S:** 74**
Gondenbrett	Rhineland–Palatinate	Goat	−	−	−	−	745 (36,247) B: **573**, S: **118**	143 (5,866) B: **42**, S: **3**	104 (3,998) B: **4**, S: 0	992 (46,111) B: **619**, S: **121**
			211 (8,775) B: 0, S: 0	1,288 (60,243) B:0, S: **1**	463 (21,389) B: **2**, S: **3**	1,402 (65,986) B: **76**, S: **66**	2,945 (134,503) B: **1,181**, S: **298**	907 (36,045) B: **96**, S: **33**	333 (10,418) B: **4**, S: **1**	7,549 (337,359) B: **1,359**, S: **402**

The total numbers of tested *Culicoides* pools, individual *Culicoides* (in brackets), and of BTV–RNA (B) and SBV–RNA (S)-positive pools (bold) from each site during the monitoring period are displayed. In addition, information on the corresponding German federal state and the species of livestock kept is provided

Some *Culicoides* species are morphologically indistinguishable, and therefore, morphological identification was initially conducted to subgenus level. Individual female *Culicoides,* regardless of their parity status, were morphologically examined and assigned to the *Avaritia* subgenus, the *Culicoides* subgenus or ‘other’, using a stereomicroscope (Leica model M205 C; Leica Microsystems GmbH, Wetzlar, Germany) and the keys of Mathieu et al. [38] and Delécolle [39]. Males and females assigned to ‘other’, were excluded from further analysis.

Up to 2,500 female *Culicoides* per trapping location and day of trapping were grouped in pools containing ≤ 50 midges of the same subgenus. Some additional pools per trapping location were stored as retention samples.

Processing of the pools and nucleic acid extraction were carried according to previously published protocols [37]. All extracts were tested by previously described real-time RT-PCRs for the presence of SBV [40], BTV and EHDV–RNA [41]. Pools which tested positive for SBV, BTV or EHDV were subsequently analyzed by PCR to determine the species present within the pool [42, 43]. For the subgenus *Avaritia*, a quantitative PCR (qPCR) was used [43], while for the subgenus *Culicoides*, a conventional PCR was employed [42]. These PCRs are capable of determining the species composition of the pool; however, they are not equipped to provide quantitative data.

To compare the spatial and temporal differences in virus circulation, the minimum infection rates (MIR) were calculated. The MIR is the number of positive pools divided by the number of tested pools multiplied by 1000.

## Results

A total of 337,359 female *Culicoides* belonging to the subgenera *Avaritia* or *Culicoides* from 14 trapping locations were sorted into 7,549 pools with an average pool size of 44.7 individuals. The majority of female *Culicoides* (n = 288,382) belonged to the subgenus *Avaritia* and sorted into 6,191 pools (82.0%) with an average pool size of 46.6. A total of 48,977 females were assigned to the subgenus *Culicoides* and placed in 1,358 pools (18.0%) with an average pool size of 36.1. The difference in the average pool size reflects the number of individuals available from each subgenus and each trapping day and trapping site.

All pools were tested by real-time RT-PCRs for the genomes of SBV, BTV and EHDV. Altogether, 1,359 pools (18.0%) tested positive for BTV–RNA and 402 pools (5.3%) for SBV–RNA (Table 1). The RNA of both, BTV and SBV was detected in 196 pools (2.6%). EHDV–RNA was not detected in any of the tested pools. None of the *Culicoides* pools collected in April were positive for viral genome. In May, only one SBV–RNA-positive pool (SBV–MIR: 0.8) was detected. In June, two pools tested positive for BTV–RNA (BTV–MIR: 4.3) and three pools for SBV–RNA (SBV–MIR: 6.5). In July, BTV–RNA was found in 76 pools (BTV–MIR: 54.2), while SBV–RNA was found in 66 pools (SBV–RNA: 47.1). In August, 1,181 pools were positive for BTV–RNA (BTV–MIR: 401.0) and 298 pools for SBV–RNA (SBV–MIR: 101.2). In September, BTV–RNA was detected in 96 pools (BTV–MIR: 105.8) and SBV–RNA in 33 pools (SBV–MIR: 36.4). In October, four pools tested positive for BTV (BTV–MIR: 12.0) and one pool for SBV (SBV–MIR: 3.0). The results of the virus screening for each monitored month and for the individual trapping sites are depicted in Fig. 1B–M and in Table 1.

BTV–RNA-positive pools were found on 11, and SBV–RNA-positive pools on 10 out of the 14 monitored farms (Table 1). At most of the monitored locations, August was the month with the highest MIR for both BTV and SBV. As demonstrated in Fig. 1C–M and Table 1, the peak of BTV circulation was observed at nine trapping sites in August and at two sites in September. For SBV, the highest circulation was observed at seven trapping sites in August, two in July and one in September.

**Fig. 2:**
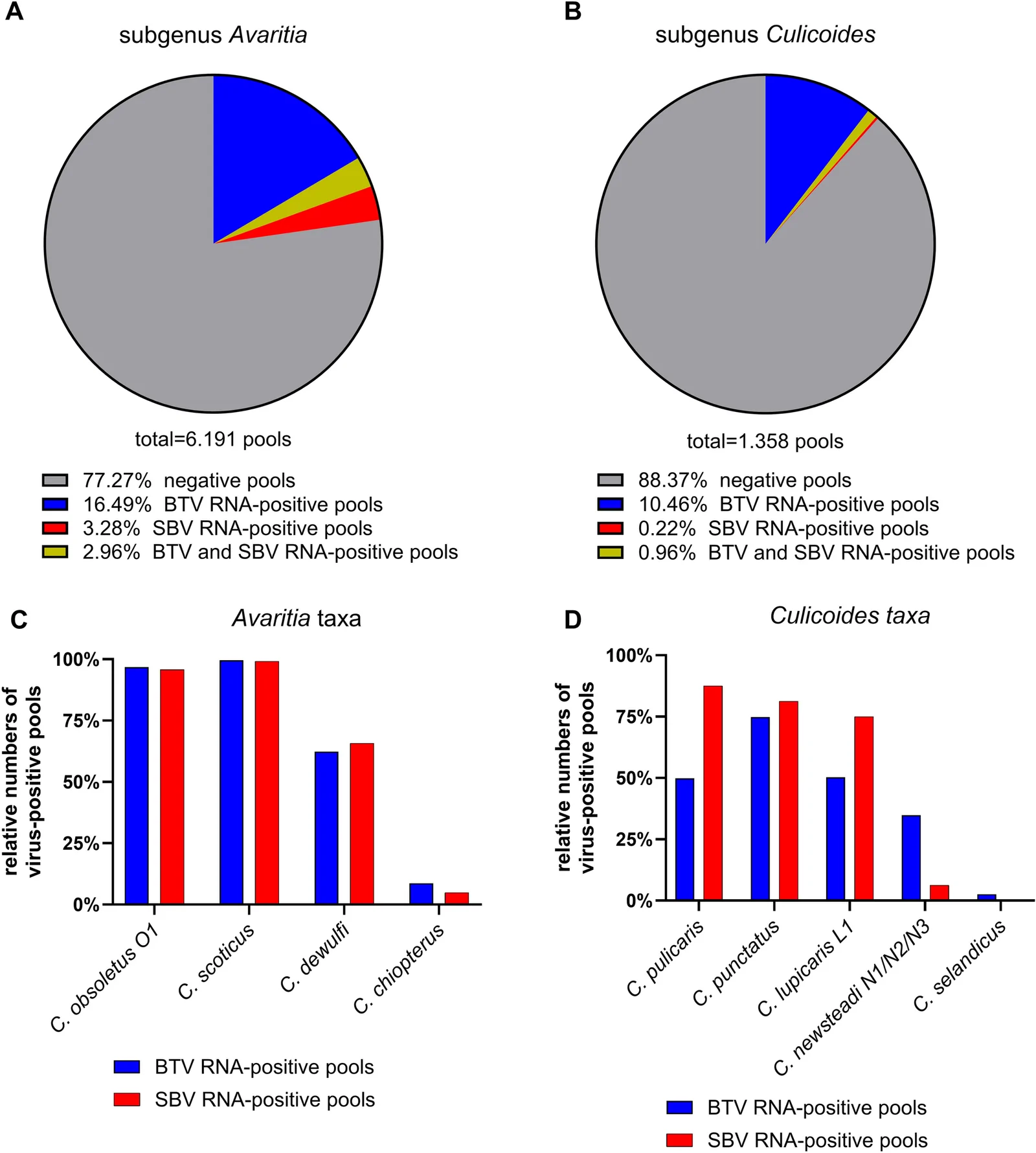
Comparison of the percentages of virus-positive pools containing species of the subgenus *Avaritia* or the subgenus *Culicoides* and specification of the species found in the virus-positive pools of both subgenera. The relative numbers of pools tested negative (gray), BTV–RNA-positive pools (blue), SBV–RNA-positive pools (red) and BTV and SBV–RNA-positive pools (yellow) of the subgenus *Avaritia* are depicted in (**A**) and equivalent pools belonging to the subgenus *Culicoides* in (**B**). The relative numbers of BTV–RNA-positive (blue) and SBV–RNA-positive (red) pools containing the individual identified species of the subgenus *Avaritia* are presented in (**C**) and the identified species of the subgenus *Culicoides* in (**D**)

In total, 1,407 pools (22.7%) out of the subgenus *Avaritia* pools were positive for BTV and/or SBV–RNA. This proportion is higher than the 158 pools (11.6%) of the subgenus *Culicoides* pools that were found to be positive for viral RNA. BTV–RNA was detected in 19.4% of the *Avaritia* pools and in 11.4% of the *Culicoides* pools (Fig. 2A, B). Furthermore, 6.2% of the *Avaritia* and 1.2% of the *Culicoides* pools tested positive for SBV–RNA (Fig. 2A, B). In both SBV- and BTV–RNA-positive *Culicoides* pools, the most prevalent taxa were *C. obsoletus* clade O1, *C. scoticus* and *C. dewulfi* of the subgenus *Avaritia* and *C. punctatus*, *C. pulicaris* and *Culicoides lupicaris* Downes & Kettle haplotype L1 of the subgenus *Culicoides* (Fig. 2C, D).

## Discussion

In this study, we monitored the presence of SBV, BTV and EHDV in *Culicoides* biting midges collected from April to October 2024, especially during the early outbreak phase of BTV-3 in north–west Germany following the initial detection in autumn 2023. This enabled us to make unique comparisons between some circulation characteristics of the enzootic SBV and the newly emerging BTV-3. Furthermore, we were able to check for EHDV and compare all three *Culicoides*-borne animal viruses.

In 2024, the *Culicoides*-monitoring program focused on the north-western German federal states. With this geographical focus, it was possible to monitor the BTV circulation during the first year of the BTV-3 outbreak in Germany, where a naïve and unvaccinated ruminant population was present. In April 2024, the utilization of an autogenous BTV-3 vaccine (ANIVAC BTV-3, San Group Biotech Germany) for the vaccination of ruminants in the affected German federal states was authorized. However, due to the observation of approximately 13% of the vaccinated animals becoming ill [44], the vaccine was used limitedly and was subsequently withdrawn. The possibility of this virus vaccine being transmitted by biting midges and the potential epidemiological consequences are not known. The actual BTV-3 vaccination campaign started in June 2024 when the Federal Ministry approved the usage of three BTV-3 specific vaccines [45], around the time when BTV cases were detected in high numbers also in other parts of Germany (over 8,900 reported cases in ruminants from June to August throughout the country, source: German animal disease reporting system TSN).

The present investigation represents the second monitoring study in Germany with BTV detection in *Culicoides* biting midges over a period of several months. The first study was conducted from April 2007 to May 2008 during the outbreak of BTV-8 [2]. Both studies have similar backgrounds: monitoring the BTV vectors in the year after the initial outbreaks in Germany. As opposed to the 2024 study, when the peak of BTV circulation in the *Culicoides* population was observed in August, in 2007, BTV-8–RNA-positive pools were detected from July to November, with a peak in October [2]. However, no direct comparison of the circulation of BTV-8 and BTV-3 in *Culicoides* caught from single trapping sites is possible, since the trapping sites differed between the studies.

Differences in virus circulation also become visible by comparing the monthly numbers of reported cases of infections in ruminants from 2007 and 2024. In both outbreak years, the majority of BTV cases in ruminants occurred from August to October (source: TSN). However, in 2007, the number of reported ruminant BTV cases peaked in September (9,269 cases) and was still much higher in October (5,254 cases) than in August (2,175 cases). In contrast, in 2024, more cases were reported in August (5,907 cases) than in September (4,813 cases) and October (1,996 cases) (source: TSN). Thus, the temporal trend in reported BTV cases among ruminants in 2024 was consistent with the BTV–MIR observed through *Culicoides* surveillance, suggesting an early increase in BTV-3 circulation and transmission.

The observed variation in the monthly occurrence of the two BTV serotypes in 2007 and 2024 may be the result of differences in environmental, climatic, and ecological factors between the studied years. Regional abundance and the ability for virus transmission of *Culicoides* are influenced by a lot of factors, such as temperature, weather, breeding conditions, population density and the availability of host species and their immune status [1, 13, 46, 47]. In both monitored outbreak years, 2007 and 2024, the viruses met with a largely naïve ruminant population, and both studies focused on the west of Germany, where BTV-8 and BTV-3 had first emerged, entering from the Netherlands. The environmental factors for the individual trapping sites such as the abundance of *Culicoides*, the breeding conditions for the immature midge stages and the climatic conditions at the trapping sites cannot be compared between the studied years, since the monitoring took place on different farms in 2 years and no information is available about the environmental conditions at the trapping sites from 2007. However, with 10.9 °C, the average temperature in Germany was higher in 2024 than with 9.9 °C in 2007 [48, 49]. Virus replication by *Culicoides* is strongly temperature-dependent [50], and higher temperatures in 2024 than in 2007 could explain the faster outbreak progression of BTV-3 in 2024. Consequently, the proportion of seroconverted ruminants was already very high by August 2024, which may have resulted in the observed decline in new BTV-3 infections in autumn 2024. In addition, the vaccination campaign, which reduced the number of naïve ruminant hosts, may have contributed to the decline in the BTV wave.

The results of the monitoring study confirm the ongoing circulation of SBV in Germany, which was already observed in studies conducted until 2023 [31, 37]. Since the first detection of SBV in Germany in 2011, cases of virus infections in ruminants have occurred annually (source: TSN), although it is likely that these numbers do not reflect the true SBV circulation. The monitoring of SBV–RNA in *Culicoides* has proven to be a suitable alternative for retrospective observation of the SBV circulation [37]. In a former monitoring study, carried out in Germany from 2019 to 2023, SBV–RNA was detected in the *Culicoides* in every year [37]. Compared to the observed SBV circulation in North Rhine–Westphalia and Lower Saxony from late September to early November 2023 with an MIR of 135.5, the detected MIR of 22.8 from September to November 2024 with only 25 SBV–RNA-positive pools out of 1,537 tested pools was lower. This decrease in SBV circulation can be well-explained by the re-circulation of SBV at high levels every 2–4 years, and the subsequent herd immunity effect, as previously described [37, 51, 52].

The seasonal occurrence of SBV in *Culicoides* in 2024 in north–west Germany is similar to the observed monthly SBV circulation from 2019 to 2022 in all of Germany [37]. In 2024, the SBV circulation peaked in August, whereas from 2019 to 2022, the highest SBV prevalence was detected in August and September [37]. Furthermore, it appears that the circulation of SBV, as well as that of BTV, within the *Culicoides* population in 2024 was dependent on the development of temperature over the monitored months (Fig. 1B). In April, when the mean temperature in the monitored federal states was at its lowest, with an average of 10.3 °C, no viruses were detected in the tested pools. As the average temperature increased from May to August 2024, the number of SBV–RNA- and BTV–RNA-positive pools increased in parallel (Fig. 1B). The peak average temperature of 19.7 °C in August corresponds with the highest BTV– and SBV–MIR (Fig. 1B). In September and October, average temperatures dropped again, and with them the number of virus-positive pools (Fig. 1B).

The MIRs in the *Culicoides* observed in 2024 in north–west Germany were higher for BTV than for SBV. However, the monthly distribution of BTV–RNA- and SBV–RNA-positive pools was very similar (Fig. 1B–M). SBV and BTV are viruses of different orders and differ in many characteristics such as duration of viremia in the mammalian hosts [22, 53]. The observed similarity of the MIR curves for BTV and SBV over the months in 2024 (Fig. 1B–M) may suggest a virus-independent effect on virus circulation in the *Culicoides* vector, supporting the theory that the overall conditions for virus transmission by *Culicoides* at the trapping sites caused the peaks in BTV and SBV circulation in August 2024. This hypothesis is supported by the fact that virus circulation differs between trapping sites, presumably due to different prevailing conditions. As demonstrated in Fig. 1C–M, the monthly occurrence of the viruses is frequently comparable for SBV and BTV when the locations are analyzed individually.

Pooled female *Culicoides* were tested regardless of their parity status and were processed as whole individuals. Specimens were not separated by parity, since parity grading based on abdominal pigmentation is known to have a considerable error rate [54], particularly in large-scale surveillance samples. The majority of both BTV–RNA- and SBV–RNA-positive pools contained *Culicoides* species of the subgenus *Avaritia,* which is not surprising, as this subgenus is more common in Germany than the subgenus *Culicoides* [2, 14]. The identification of the *Avaritia* and *Culicoides* taxa included in the BTV and SBV–RNA-positive pools produced similar results (Fig. 2C, D). This suggests that various *Culicoides* taxa can transmit these viruses, regardless of their characteristics, equally well under suitable environmental conditions. However, it is more likely that the observed similarity in the BTV and SBV–RNA-positive taxa is attributable to the frequency of occurrence of the different species on the monitored farms in Germany. *Culicoides obsoletus* clade O1*, C. scoticus* and *C. dewulfi* as well as *C. punctatus, C. pulicaris* and *C. lupicaris* haplotype L1 are the most common *Culicoides* taxa in Germany, and are, therefore, also most frequently found in the virus-positive pools. To a lesser extent, virus genome was detected in the *Avaritia* pools containing the species *C. chiopterus* and in *Culicoides* pools with the taxa *Culicoides newsteadi* Austen haplotypes N1, N2 and N3 (Fig. 2C, D). Furthermore, four pools containing the subgenus *Culicoides* species *Culicoides selandicus* Nielsen, Kristensen & Pape, among others, tested positive for BTV–RNA (Fig. 2D). However, the fact that certain *Culicoides* species were found frequently in virus-positive pools does not itself demonstrate vector competence of those species. Overall, no conclusion on vector competence can be drawn when viral RNA is detected in pooled samples. Since most of the virus-positive pools contained more than one taxon, it is not possible to ascertain which one was infected with the virus. Furthermore, the *Culicoides* were examined as whole individuals. It is only possible to determine whether a field-collected haematophagous insect can transmit a virus with certainty if the virus is detected in the salivary glands [16]. While SBV transmission has been demonstrated for *C. obsoletus* and *C. scoticus* [4, 5], and is most likely for *C. dewulfi* and *C. chiopterus* [3], no information on the vector competence is available for the taxa of the subgenus *Culicoides*. By contrast, some taxa of the subgenus *Culicoides* seem to be competent vectors of BTV, such as *C. punctatus*, *C. pulicaris*, *Culicoides impunctatus* Goetghebuer and *C. newsteadi* [6–10, 55, 56]. Information is also lacking regarding the vector competence of *C. selandicus* for BTV.

A limitation of this study is that the *Culicoides* were pooled according to their subgenus and not identified to species level. This might have given insights into the relative importance of each species as a vector. However, as already mentioned above, no final conclusion can be drawn by the examination of *Culicoides* as whole individuals. Another limitation of this study is the inconsistent availability of *Culicoides* samples over the monitored season on the individual trapping sites (Fig. 1C–M). In particular, samples from the trapping sites of Neukirchen, Gondenbrett and Balve were available from August onward. Therefore, no statement can be made about the peak of virus circulation on those trapping sites with inconsistent sample availability. As is common in large-scale national monitoring programs, there are several causes of gaps in the data: some trap sites were set up later in the season; no *Culicoides* were caught at certain sites during certain periods; and some samples were not suitable for testing.

Altogether, we observed a similar distribution of *Culicoides* taxa in the virus-positive pools and a similar seasonal circulation pattern of the enzootic SBV and the newly introduced BTV-3 in the *Culicoides* population in western Germany in 2024. Though not all factors that influence the transmission of *Culicoides*-borne viruses are yet fully understood, they seem to affect the transmission of viruses of different orders and characteristics, such as BTV and SBV, in a similar way.

## Conclusions

The results of this monitoring study demonstrate a high level of BTV circulation in the *Culicoides* population in western Germany during the BTV-3 outbreak in 2024. In contrast, EHDV was not detected in any of the tested *Culicoides* pools, which is consistent with the absence of ruminant cases of EHDV in Germany. Furthermore, we confirmed the ongoing enzootic circulation of SBV in the *Culicoides* population in western Germany, highlighting the importance of continuous *Culicoides* monitoring. In addition, the study shows that further research into the factors influencing *Culicoides*-borne virus transmission is essential if measures are to be taken to control the spread of viruses.

## Data Availability

The datasets generated and analysed during the current study are available in the Zenodo repository, https://doi.org/10.5281/zenodo.21486935.

## Abbreviations

SBV: Schmallenberg virus
BTV: Bluetongue virus
EHDV: Epizootic hemorrhagic disease virus
MIR: Minimum infection rate
PCR: Polymerase chain reaction
RT-PCR: Reverse transcriptase polymerase chain reaction
TSN: Tierseuchen-Nachrichtensystem (German animal disease reporting system)
Cq value: Quantification cycle value

## References

[1] Mellor PS, Boorman J, Baylis M. *Culicoides* biting midges: their role as arbovirus vectors. Annu Rev Entomol. 2000;45:307–40. 10.1146/annurev.ento.45.1.307 10761580

[2] Hoffmann B, Bauer B, Bauer C, Bätza HJ, Beer M, Clausen PH, et al. Monitoring of putative vectors of bluetongue virus serotype 8, Germany. Emerg Infect Dis. 2009;15:1481–4. 10.3201/eid1509.090562 PMC2819873 19788820

[3] De Regge N, De Deken R, Fassotte C, Losson B, Deblauwe I, Madder M, et al. *Culicoides* monitoring in Belgium in 2011: analysis of spatiotemporal abundance, species diversity and Schmallenberg virus detection. Med Vet Entomol. 2011;29:263–75. 10.1111/mve.12109 25761054

[4] Pagès N, Talavera S, Verdún M, Pujol N, Valle M, Bensaid A, et al. Schmallenberg virus detection in *Culicoides* biting midges in Spain: first laboratory evidence for highly efficient infection of *Culicoides* of the Obsoletus complex and *Culicoides imicola*. Transbound Emerg Dis. 2018;65:e1–6. 10.1111/tbed.12653 28474491

[5] Balenghien T, Pagès N, Goffredo M, Carpenter S, Augot D, Jacquier E, et al. The emergence of Schmallenberg virus across *Culicoides* communities and ecosystems in Europe. Prev Vet Med. 2014;116:360–9. 10.1016/j.prevetmed.2014.03.007 24698329

[6] Carpenter S, McArthur C, Selby R, Ward R, Nolan DV, Mordue Luntz AJ, et al. Experimental infection studies of UK *Culicoides* species midges with bluetongue virus serotypes 8 and 9. Vet Rec. 2008;163:589–92. 10.1136/vr.163.20.589 19011244

[7] Paslaru AI, Mathis A, Torgerson P, Veronesi E. Vector competence of pre-alpine *Culicoides* (Diptera: Ceratopogonidae) for bluetongue virus serotypes 1, 4 and 8. Parasit Vectors. 2018;11:466. 10.1186/s13071-018-3050-y PMC6090685 30103803

[8] Federici V, Goffredo M, Mancini G, Quaglia M, Santilli A, Di Nicola F, et al. Vector competence of Italian populations of *Culicoides* for some bluetongue virus strains responsible for recent northern African and European outbreaks. Viruses. 2019; 11:941. 10.3390/v11100941. PMC6832517 31614799

[9] Carpenter S, Lunt HL, Arav D, Venter GJ, Mellor PS. Oral susceptibility to bluetongue virus of *Culicoides* (Diptera: Ceratopogonidae) from the United Kingdom. J Med Entomol. 2006;43:73–8. 10.1093/jmedent/43.1.73 16506450

[10] Purse B, Carpenter S, Venter G, Bellis G, Mullens B. Bionomics of temperate and tropical *Culicoides* midges: knowledge gaps and consequences for transmission of *Culicoides*-borne viruses. Annu Rev Entomol. 2015;60:373–92. 10.1146/annurev-ento-010814-020614 25386725

[11] Maurer LM, Paslaru A, Torgerson PR, Veronesi E, Mathis A. Vector competence of *Culicoides* biting midges from Switzerland for African horse sickness virus and epizootic haemorrhagic disease virus. Schweiz Arch Tierheilkd. 2022;164:66–70. 10.17236/sat00337 34983740

[12] Savini G, Afonso A, Mellor P, Aradaib I, Yadin H, Sanaa M, et al. Epizootic haemorrhagic disease. Res Vet Sci. 2011;91:1–17. 10.1016/j.rvsc.2011.05.004 21665237

[13] Werner D, Groschupp S, Bauer C, Kampen H. Breeding habitat preferences of major *Culicoides* species (Diptera: Ceratopogonidae) in Germany. Int J Environ Res Public Health. 2020;17:5000. 10.3390/ijerph17145000. PMC7400431 32664561

[14] Groschupp S, Kampen H, Werner D. Occurrence of putative *Culicoides* biting midge vectors (Diptera: *Ceratopogonidae*) inside and outside barns in Germany and factors influencing their activity. Parasit Vectors. 2023;16:307. 10.1186/s13071-023-05920-z PMC10472570 37653421

[15] Sick F, Beer M, Kampen H, Wernike K. *Culicoides* biting midges-underestimated vectors for arboviruses of public health and veterinary importance. Viruses. 2019. 10.3390/v11040376. PMC6520762 31022868

[16] Mellor PS. Replication of arboviruses in insect vectors. J Comp Pathol. 2000;123:231–47. 10.1053/jcpa.2000.0434 11041993

[17] Hoffmann B, Scheuch M, Höper D, Jungblut R, Holsteg M, Schirrmeier H, et al. Novel orthobunyavirus in cattle, Europe, 2011. Emerg Infect Dis. 2011;18:469–72. 10.3201/eid1803.111905 PMC3309600 22376991

[18] Beer M, Conraths FJ, van der Poel WH. Schmallenberg virus’–a novel orthobunyavirus emerging in Europe. Epidemiol Infect. 2013;141:1–8. 10.1017/S0950268812002245 PMC9152057 23046921

[19] Wernike K, Fischer L, Holsteg M, Aebischer A, Petrov A, Marquart K, et al. Serological screening in wild ruminants in Germany, 2021/2022: no evidence of SARS-CoV-2, bluetongue virus or pestivirus spread but high seroprevalences against Schmallenberg virus. Transbound Emerg Dis. 2021;69:e3289–96. 10.1111/tbed.14600 PMC9348064 35585653

[20] Laloy E, Riou M, Barc C, Belbis G, Bréard E, Breton S, et al. Schmallenberg virus: experimental infection in goats and bucks. BMC Vet Res. 2015;11:221. 10.1186/s12917-015-0516-4 PMC4546222 26297244

[21] van den Brom R, Luttikholt SJ, Lievaart-Peterson K, Peperkamp NH, Mars MH, van der Poel WH, et al. Epizootic of ovine congenital malformations associated with Schmallenberg virus infection. Tijdschr Diergeneeskd. 2012;137:106–11. 22393844

[22] Wernike K, Hoffmann B, Bréard E, Bøtner A, Ponsart C, Zientara S, et al. Schmallenberg virus experimental infection of sheep. Vet Microbiol. 2013;166:461–6. 10.1016/j.vetmic.2013.06.030 23972950

[23] Wernike K, Silaghi C, Nieder M, Pfeffer M, Beer M. Dynamics of Schmallenberg virus infection within a cattle herd in Germany, 2011. Epidemiol Infect. 2014;142:1501–4. 10.1017/S0950268813002525 PMC9151231 24128891

[24] Herder V, Wohlsein P, Peters M, Hansmann F, Baumgärtner W. Salient lesions in domestic ruminants infected with the emerging so-called Schmallenberg virus in Germany. Vet Pathol. 2012;49:588–91. 10.1177/0300985812447831 22610033

[25] Wernike K, Holsteg M, Schirrmeier H, Hoffmann B, Beer M. Natural infection of pregnant cows with Schmallenberg virus--a follow-up study. PLoS ONE. 2014;9:e98223. 10.1371/journal.pone.0098223 PMC4031169 24853555

[26] Subhadra S, Sreenivasulu D, Pattnaik R, Panda BK, Kumar S. Bluetongue virus: past, present, and future scope. J Infect Dev Ctries. 2023;17:147–56. 10.3855/jidc.16947 36897898

[27] Gondard M, Postic L, Garin E, Turpaud M, Vorimore F, Ngwa-Mbot D, et al. Exceptional bluetongue virus (BTV) and epizootic hemorrhagic disease virus (EHDV) circulation in France in 2023. Virus Res. 2023;350:199489. 10.1016/j.virusres.2024.199489 PMC11565556 39471970

[28] Lorusso A, Cappai S, Loi F, Pinna L, Ruiu A, Puggioni G, et al. Epizootic hemorrhagic disease virus serotype 8, Italy, 2022. Emerg Infect Dis. 2022;29:1063–5. 10.3201/eid2905.221773 PMC10124640 37081599

[29] Saminathan M, Singh KP, Khorajiya JH, Dinesh M, Vineetha S, Maity M, et al. An updated review on bluetongue virus: epidemiology, pathobiology, and advances in diagnosis and control with special reference to India. Vet Q. 2020;40:258–321. 10.1080/01652176.2020.1831708 PMC7655031 33003985

[30] Santman-Berends I, van den Brink K, Dijkstra E, van Schaik G, Spierenburg MAH, van den Brom R. The impact of the bluetongue serotype 3 outbreak on sheep and goat mortality in the Netherlands in 2023. Prev Vet Med. 2024;231:106289. 10.1016/j.prevetmed.2024.106289 39126984

[31] Voigt A, Kampen H, Heuser E, Zeiske S, Hoffmann B, Höper D, et al. Bluetongue virus serotype 3 and Schmallenberg virus in *Culicoides* biting midges, western Germany, 2023. Emerg Infect Dis. 2023;30:1438–41. 10.3201/eid3007.240275 PMC11210651 38916645

[32] Spedicato M, Profeta F, Thabet S, Teodori L, Leone A, Portanti O, et al. Experimental infection of cattle, sheep, and goats with the newly emerged epizootic hemorrhagic disease virus serotype 8. Vet Ital. 2023;59. 10.12834/VetIt.3433.23112.1 38117055

[33] MacLachlan NJ. Bluetongue: pathogenesis and duration of viraemia. Vet Ital. 2004;40:462–7. 20422570

[34] Maclachlan NJ, Zientara S, Savini G, Daniels PW. Epizootic haemorrhagic disease. Rev Sci Tech. 2015;34:341–51. 10.20506/rst.34.2.2361 26601439

[35] Darpel KE, Batten CA, Veronesi E, Shaw AE, Anthony S, Bachanek-Bankowska K, et al. Clinical signs and pathology shown by British sheep and cattle infected with bluetongue virus serotype 8 derived from the 2006 outbreak in northern Europe. Vet Rec. 2006;161:253–61. 10.1136/vr.161.8.253 17720961

[36] Worwa G, Hilbe M, Ehrensperger F, Chaignat V, Hofmann MA, Griot C, et al. Experimental transplacental infection of sheep with bluetongue virus serotype 8. Vet Rec. 2009;164:499–500. 10.1136/vr.164.16.499 19377090

[37] Zeiske S, Kampen H, Sick F, Dähn O, Voigt A, Heuser E, et al. Monitoring of Schmallenberg virus, bluetongue virus and epizootic haemorrhagic disease virus in biting midges in Germany 2019–2023. Parasit Vectors. 2025;18:262. 10.1186/s13071-025-06913-w PMC12229043 40618098

[38] Mathieu B, Cêtre-Sossah C, Garros C, Chavernac D, Balenghien T, Carpenter S, et al. Development and validation of IIKC: an interactive identification key for *Culicoides* (Diptera: Ceratopogonidae) females from the Western Palaearctic region. Parasit Vectors. 2012;5:137. 10.1186/1756-3305-5-137 PMC3483010 22776566

[39] Delécolle J-C. 1985. Nouvelle contribution à l'étude systématique et iconographique des espèces du genre *Culicoides*, (Diptéra):(Cératopogonidae) du Nord-Est de la France: France University of Strasbourg

[40] Bilk S, Schulze C, Fischer M, Beer M, Hlinak A, Hoffmann B. Organ distribution of Schmallenberg virus RNA in malformed newborns. Vet Microbiol. 2012;159:236–8. 10.1016/j.vetmic.2012.03.035 22516190

[41] Wernike K, Hoffmann B, Beer M. Simultaneous detection of five notifiable viral diseases of cattle by single-tube multiplex real-time RT-PCR. J Virol Methods. 2015;217:28–35. 10.1016/j.jviromet.2015.02.023 25746154

[42] Dähn O, Werner D, Mathieu B, Kampen H. Development of conventional multiplex PCR assays for the identification of 21 west Palaearctic biting midge taxa (Diptera: Ceratopogonidae) belonging to the *Culicoides* subgenus *Culicoides,* including recently discovered species and genetic variants. Diversity. 2023;15:699.

[43] Dähn O, Werner D, Mathieu B, Kampen H. Large-scale cytochrome c oxidase subunit I gene data analysis for the development of a multiplex polymerase chain reaction test capable of identifying biting midge vector species and haplotypes (Diptera: Ceratopogonidae) of the *Culicoides* subgenus *Avaritia* Fox, 1955. Genes. 1955;15:323. 10.3390/genes15030323 PMC10969821 38540382

[44] Reviriego-Gordejo F, Minne D, Claassen I, Cavitte JC, Bouma A, Debaere O, et al. Preparedness and response to emerging veterinary disease outbreaks - a meeting report. Biologicals. 2026;93:101873. 10.1016/j.biologicals.2026.101873 41534219

[45] Bundesministerium der Justiz und für Verbraucherschutz. Zweite Verordnung über bestimmte Impfstoffe zum Schutz vor der Blauzungenkrankheit (BTV-3-ImpfgestattungsV). 2024.https://www.gesetze-im-internet.de/btv-3-impfgestattungsv/BJNR0B50A0024.html. Last accessed 03.10.2026.

[46] Silbermayr K, Hackländer K, Doscher C, Koefer J, Fuchs K. 2011. A spatial assessment of *Culicoides* spp. distribution and bluetongue disease risk areas in Austria. Berl Munch Tierarztl Wochenschr. 124:228–35. 22059294

[47] Carpenter M, Kopanke J, Lee J, Rodgers C, Reed K, Sherman TJ, et al. Evaluating temperature effects on bluetongue virus serotype 10 and 17 coinfection in Culicoides sonorensis. Int J Mol Sci. 2024. 10.3390/ijms25053063. PMC10932384 38474308

[48] Klimastatusbericht 2007. 2008. https://www.dwd.de/DE/leistungen/klimastatusbericht/publikationen/ksb_2007.html. Last accessed 11.06.2026.

[49] Klimastatusbericht 2024. 2025. https://www.dwd.de/DE/leistungen/klimastatusbericht/publikationen/ksb_2024.pdf?__blob=publicationFil. blob=publicationFil. Last accessed 11.06.2026

[50] Carpenter S, Wilson A, Barber J, Veronesi E, Mellor P, Venter G, et al. Temperature dependence of the extrinsic incubation period of orbiviruses in *Culicoides* biting midges. PLoS ONE. 2011;6:e27987. 10.1371/journal.pone.0027987 PMC3220716 22125649

[51] Larska M. Schmallenberg virus: a cyclical problem. Vet Rec. 2018;183:688–9. 10.1136/vr.k5109 30523184

[52] Wernike K, Beer M. Re-circulation of Schmallenberg virus, Germany, 2019. Transbound Emerg Dis. 2020;67:2290–5. 10.1111/tbed.13592 32320536

[53] Katz JB, Gustafson GA, Alstad AD, Adler KA, Moser KM. Colorimetric diagnosis of prolonged bluetongue viremia in sheep, using an enzyme-linked oligonucleotide sorbent assay of amplified viral nucleic acids. Am J Vet Res. 1993;54:2021–6. 8116932

[54] Harrup LE, Purse BV, Golding N, Mellor PS, Carpenter S. Larval development and emergence sites of farm-associated *Culicoides* in the United Kingdom. Med Vet Entomol. 2013;27:441–9. 10.1111/mve.12006 23458570

[55] Jennings DM, Mellor PS. The vector potential of British *Culicoides* species for bluetongue virus. Vet Microbiol. 1988;17:1–10. 10.1016/0378-1135(88)90074-0 2845631

[56] Foxi C, Delrio G, Falchi G, Marche MG, Satta G, Ruiu L. Role of different *Culicoides* vectors (Diptera: Ceratopogonidae) in bluetongue virus transmission and overwintering in Sardinia (Italy). Parasit Vectors. 2016;9:440. 10.1186/s13071-016-1733-9 PMC4977893 27502924

